# Novel formulation with essential oils as a potential agent to minimize African swine fever virus transmission in an *in vivo* trial in swine

**DOI:** 10.14202/vetworld.2021.1853-1866

**Published:** 2021-07-20

**Authors:** Haig Yousef Babikian, Rajeev Kumar Jha, Quang Lam Truong, Lan Thi Nguyen, Yusef Babikyan, Hoa Thi Nguyen, Thanh Long To, Ali Agus

**Affiliations:** 1 Department of Research and Development, PT. Rhea Natural Sciences, Indonesia; 2 Key laboratory of Veterinary Biotechnology, Faculty of Veterinary Medicine, Vietnam National University of Agriculture, Hanoi, Vietnam; 3 Department of Technical Research and Development, PT. Central Proteina Prima, Jakarta, Indonesia; 4 Faculty of Animal Science, University of Gajah Mada, Yogyakarta, Indonesia

**Keywords:** African swine fever virus, *In vivo* trials, intramuscular challenge, natural oil blend formulation, Swine

## Abstract

**Background and Aim:**

African swine fever (ASF) is currently the most prevalent disease in swine. The disease is spreading throughout primary swine-producing countries with heavy losses in population and revenue. To date, no successful vaccines or medications have been reported. This study aimed to design and develop a blend of natural essential oils and test its efficacy against the ASF virus (ASFV) in swine.

**Materials and Methods:**

We attempted to develop a natural oil blend formulation (NOBF) and determine its efficacy against the ASFV. This study follows on from a previously published *in vitro* study that reported that the NOBF has anti-ASFV properties. A study was designed using 21 healthy piglets of triple-cross (Landrace + Yorkshire + Durok) crossbred pathogen-free pigs with an average weight of 15 kg. The study consisted of NOBF-incubated, NOBF, positive control, and negative control groups. The NOBF groups were administered NOBF (80 mL/ton mixed in drinking water) beginning 10 days before the challenge and continuing throughout the experiment. The positive and negative control pigs consumed regular drinking water. The pigs were challenged by a sublethal dose of pure isolate ASFV strain Vietnam National University of Agriculture-ASFV-L01/HN/04/19 inoculation with 10^3.5^ HAD50/dose through the intramuscular route. There were sic pigs in each group, three pigs directly IM challenged, and three pigs were considered cohoused pigs.

**Results:**

Both challenged (three) and cohoused (three) pigs in the positive control showed clinical signs of ASFV infection, as detected by real-time polymerase chain reaction (RT-PCR) in blood samples, oral swabs, and feces. There was 100% cumulative mortality, that is, both challenged and contact pigs died in the positive control group on day 20 of infection. No signs of infection or mortality were observed in the NOBF-incubated group. The challenged pigs in the NOBF-direct challenge group showed clinical signs and mortality, whereas no clinical signs or symptoms occurred in the cohoused pigs. The immunoglobulin G (IgG) level of the contact pigs was the highest in the treatment group and the lowest in the positive control group. The IgM level of the contact pigs in the treatment groups was the lowest, whereas that of the positive control was the highest. The RT-PCR test showed that the ASFV was deactivated in the NOBF-incubated group. The challenged and contact pigs of the positive control group had high Ct values. The challenged pigs of the NOBF group had high Ct values, whereas the contact pigs from the same group and those of the negative control were negative for the ASFV, determined by PCR, in all samples. The comparison of the challenged groups showed that the appearance of the virus was delayed by at least 2 days in the NOBF group compared to the positive control group.

**Conclusion:**

The results showed that NOBF can prevent the spread of the ASFV in a population. Moreover, NOBF can enhance the pig humoral immune system by enhancing IgG levels and reducing IgM levels. This study successfully demonstrated that NOBF is an anti-ASFV agent, which prevents horizontal transmission and enhances pig humoral immunity.

## Introduction

The African swine fever (ASF) virus (ASFV) is deadly to pigs but harmless to humans [1]. ASF is one of the most severe viral diseases affecting pigs worldwide [2-5]. It is considered a “notifiable disease” by the Office International des Epizooties (International Office of Epizootics [OIE] of the World Health Organization because of its high mortality rate of up to 100% [6-8]. ASF causes acute hemorrhagic fever in domestic pigs and often results in significant economic losses because of the high rates of illness and death associated with the disease [8]. The introduction of ASFV into Denmark could result in losses of US$12 million in direct costs and US$349 million in exports. In 2011, the ASFV cost Russia US$267 million [9].

ASF outbreaks in China from August 1, 2018, to January 1, 2019, occurred with a directional trend from northeast to southwest. A spatiotemporal cluster was detected in northeast China. Based on the risk analysis conducted in this study, pig density was identified as the most critical predictor of ASF outbreaks. These results contribute to the development of more effective ASF prevention and control strategies in China and other parts of the world that are at risk [2]. In addition, ASF has appeared in all 63 provinces of Vietnam, killing more than 5.6 million pigs (more than 20% of total pigs), and pork production has decreased by 8.3%, mainly affecting small-scale farms [6].

Symptoms of ASFV include high fever, decreased appetite and weakness, difficulty in standing, and red or blue blotches on the skin (particularly around the ears and snout). Additional symptoms, such as miscarriage, stillbirths, and weak litter, can occur in sows with ASF [6,10,11]. Other symptoms, such as diarrhea, vomiting, and difficulty in breathing or coughing, can occur with disease progression [6]. The ASFV causes this disease and is a large, enveloped, structurally complex DNA virus with icosahedral morphology and an average diameter of 200 nm. This virus is the only member of the *Asfarviridae* family. The capacity of the ASFV to persist in its natural hosts and domestic pigs recovered from infection and carrying low-virulence isolates shows that it has effective mechanisms to evade host defense systems.

There are two ways to approach the protection of a population: Enhance protection so that the body can defend against the pathogen and create a clean and bio-secured environment. The role of essential oils is crucial and widely known in both protections as antiviral components [12] and in biosecurity measures as disinfectants. Natural essential oils are a mixture of complex compounds, and their chemical compositions and concentrations make them unique. Natural oil supplements enhance food digestibility and immunity as well as maintain gut health. Natural essential oils, terpenes, and phenylpropenes have two major classes of compounds. The antimicrobial action of any terpene is fairly effective in the presence of the hydroxyl group of the phenolic terpenoids and delocalized electrons [12]. Phenylpropenes have antimicrobial, anti-inflammatory, sedative, and analgesic properties [13]. The detailed rational Gas chromatography-mass Spectrometry reports of the blend formulation are described in detail in a previous study [14].

Natural immunomodulators are considered an ideal approach because of their abundance, easy processing, non-residual effects, and high efficacy against different diseases. A formulation was developed by blending three natural oils, *Eucalyptus globulus*, *Pinus sylvestris*, and *Lavandula latifolia*, with antiviral properties. Cineole, a significant component of eucalyptus oil, has potent anti-inflammatory and antimicrobial properties [13] and is used to treat primary viral infections of the respiratory tract [15]. Linalool, a significant lavender oil component, has antiviral activity [16]. Isobornyl acetate extracted from pine oil has antimicrobial properties [15]. The evaluation of the *in vitro* antiviral activities of natural substances is based mainly on the inhibition of cytopathic effects, reduction or inhibition of plaque formation, and reduction in virus yield [17].

The developed natural oil blend formulation (NOBF) was tested against the ASFV in pigs in the present study. We assessed the immune development and protection against viruses in pigs and identified the reduced incidence of horizontal transmission.

## Materials and Methods

### Ethical approval

The Ministry of Agriculture and Rural Development, approved by the Vietnam National University of Agriculture (VNUA) Animal Care and Use Committee, granted permission for this trial. ARRIVE guidelines were followed [18].

### Study period and location

This study was conducted from December 2019 to February 2020. The trial was conducted in collaboration with the PT Rhea Natural Sciences, Indonesia, and the PT Central Proteina Prima, Indonesia, in collaboration with the Centre of Research in Agriculture and Fisheries (CeRAF), Vietnam, and the VNUA, Vietnam. The trial was conducted at the VNUA, Hanoi. The animals were housed and used in isolated areas at the Biosecurity Animal Facility Center of the VNUA.

### Animals

#### NOBF development

A formulation was developed by mixing an essential oil blend using three oils: *E. globulus*, *P. sylvestris*, and *L. latifolia*, at a determined concentration. The phytochemical constituents of NOBF were analyzed using gas chromatography-mass spectrometry with stationary phase nonpolar columns, which led to the identification of 168 different compounds from n-hexane-extracted oil samples. The major constituent of *E. globulus* oil is 1,8-cineole (85%), and moderate amounts of α-pinene (2.6%), p-cymene (2.7%), aromadendrene, cuminaldehyde, globulol, and pinocarveol are also present. The major constituents of *P. sylvestris* oil include 50-97% monoterpene hydrocarbons, such as α-pinene, with lesser amounts of 3-carene, dipentene, b-pinen, D-limonene, α-terpinene, g-terpinene, cis-b-ocimene, myrcene, camphene, sabinene, and terpinolene. The major constituents of *L. latifolia* oil include 37 different compounds, including 1R-à-pinene, bicycle [2.2.1] heptane, 2,2-dimethyl-5-methylene, and tricycle [2.2. 1.0 (2,6)] heptane, 1,7,7-trimethyl. Essential oils, *E. globulus*, *P. sylvestris*, and *L. latifolia* were obtained from vendors who comply with the strictest industry practices: Demeter Agro Research and Improvements Pty Ltd., New Directions Australia Pty Ltd., and Australian Botanical Products Pty Ltd. Each natural oil was obtained through the steam distillation process and was thoroughly checked for quality and chemical composition based on European Pharmacopeia. After the natural oils were declared to pass the quality check, *E. globulus*, *P. sylvestris*, and *L. latifolia* were mixed in equal quantities (1:1:1) to form the NOBF. Mixing and blending were performed in a stainless-steel ribbon mixer at 25-27°C.

#### Toxicity test of the NOBF

The formulation was pretested on animals for toxicity and tolerance levels in pigs. The toxicity level of NOBF was tested by intramuscular (IM) injection of different doses, such as 0.1%, 1%, and 2% of body weight of NOBF in live pigs. The doses were determined based on the outcomes obtained from an *in vitro* trial [14]. The activity and behavior of the pigs were observed for 2 weeks. The toxicity level of NOBF was determined by visually observing the number of porcine alveolar macrophages (PAMs) present or absent, the number of dead or alive cells, and by conducting real-time polymerase chain reaction (RT-PCR), as described in our previous study [14].

#### ASFV preparation and challenge

ASFV strain VNUA01/04.2019 was adapted to grow in PAMs and was further passaged in PAMs, and the stock used was obtained after the 15^th^ passage. The predetermined sublethal dose VNUA-ASFV-L01/HN/04/19 injected with 10^3.5^ HAD50/dose of ASFV was collected and injected into the experimental pigs by the IM challenge method.

#### In vivo trial design

Twenty-one piglets (six per group) that were evenly distributed in size, weight (13-17 kg), and sex (random) were procured from a bio-secured hatchery. The piglets were screened for known pathogens, including ASFV, foot and mouth disease virus (FMDV), porcine reproductive and respiratory syndrome virus (PRRSV), classical swine fever virus (CSFV), and piglets negative for ASFV immunoglobulin G (IgG). The piglets were acclimatized for 3 days in the experimental facility. The pelleted CP-Vietnam feed was provided twice daily. The feeding rate in week 1 was 800 g/pig/day, whereas from week 2 onward, it was 1 kg/pig/day. After acclimation, the pigs were weighed and evenly distributed to each experimental group. Six pigs were selected for each of the treatment and positive control groups, and three pigs were selected for the negative control group. The six pigs in each treatment and positive control group were subgrouped into challenged, contact, or cohoused groups (Table-1). NOBF (80 ppm) was mixed in the drinking water starting from 10 days before the challenge and continued throughout the experiment daily. The animals were screened by RT-PCR for various pathogens and found negative as described in Table-2.

**Table-1:** Experimental designs for an in vivo challenge trial using NOBF against African swine fever virus.

Groups	No. of pigs	Marked	Treatment
Group 1: Negative control	3	Not challenged	+No virus inoculation
Group 2: Positive control ASF virus direct IM challenge	3	Challenged pigs (ASF virus direct IM challenge)	+VNUA-ASFV-L01/HN/04/19 inoculation with 10^3.5^ HAD50/dose +Direct challenge of virus +Commercial disinfectant alternate days
	3	Cohoused pigs	+No virus inoculation +Commercial disinfectant alternate days
Group 3: Treatment NOBF as water supplement	3	Challenged pigs ASF virus direct IM challenge)	+ VNUA-ASFV-L01/HN/04/19 inoculation with 10^3.5^ HAD50/dose +Direct challenge of virus +NOBF at a dose of 80 ppm applied in drinking water daily
	3	Cohoused pigs	+No virus inoculation +NOBF at a dose of 80 ppm applied in drinking water daily
Group 4: Treatment NOBF as water supplement	3	Challenged pigs ASF virus incubated in NOBF and then IM challenge)	+VNUA-ASFV-L01/HN/04/19 inoculation with 10^3.5^ HAD50/dose +Virus incubated with NOBF challenged to pigs +NOBF at a dose of 80 ppm applied in drinking water daily
	3	Cohoused pigs	+No virus inoculation +NOBF at a dose of 80 ppm applied in drinking water daily

ASFV=African swine fever virus, NOBF=Natural oil blend formulation

**Table-2:** Pathogen screening of experimental pigs before in vivo trials.

Groups		No. of pigs tested	Real-time PCR results				ELISA
	
			ASFV	FMDV	PRRSV	CSFV	ASFV IgG
1	Negative control	3	negative	negative	negative	negative	negative
2	Positive control	6	negative	negative	negative	negative	negative
3	Treatment group 1 (NOBF) with direct challenge	6	negative	negative	negative	negative	negative
4	Treatment group 2 (NOBF) with incubated challenge	6	negative	negative	negative	negative	negative

ASFV=African swine fever virus, NOBF=Natural oil blend formulation, FMDV=Foot and mouth disease virus, PRRSV=Porcine reproductive and respiratory syndrome virus, CSFV=Classical swine fever virus

On day 11 of NOBF supplement intake, pigs were checked for their behavior and wellness and were found to be healthy. Then, 2 mL of a sublethal dose of ASFV (VNUA-ASFV-L01/HN/04/19 of 10^3.5^ hemosorption [HAD50]/dose) was IM injected into three pigs of each of the treatment and positive control pig groups, which were grouped as the challenged group. Three other pigs, called “contact pigs,” were cohoused with the challenged pigs.

The experimental pigs were monitored daily for body temperature and clinical signs (Table-3). Blood, oral, and fecal swab samples were collected to test the ASF viral load using RT-PCR every alternate day. Necropsy was immediately performed if the animal died during the day to observe the pathological lesions.

**Table-3:** Checklist of the clinical signs checked in individual pigs in each group after an in vivo challenge trial using NOBF against African swine fever virus.

No.	Clinical signs	No. of pigs in positive control	No. of pigs in treatment	No. of pigs in negative control
1	High fever	No. of pigs with symptoms/No. of pigs observed	No. of pigs with symptoms/No. of pigs observed	No. of pigs with symptoms/No. of pigs observed
2	Depression	No. of pigs with symptoms/No. of pigs observed	No. of pigs with symptoms/No. of pigs observed	No. of pigs with symptoms/No. of pigs observed
3	Loss of appetite and reflexes	No. of pigs with symptoms/No. of pigs observed	No. of pigs with symptoms/No. of pigs observed	No. of pigs with symptoms/No. of pigs observed
4	Vomiting	No. of pigs with symptoms/No. of pigs observed	No. of pigs with symptoms/No. of pigs observed	No. of pigs with symptoms/No. of pigs observed
5	Respiratory distress	No. of pigs with symptoms/No. of pigs observed	No. of pigs with symptoms/No. of pigs observed	No. of pigs with symptoms/No. of pigs observed
6	Cyanosis on the edges of the ears, tail and legs	No. of pigs with symptoms/No. of pigs observed	No. of pigs with symptoms/No. of pigs observed	No. of pigs with symptoms/No. of pigs observed
7	Hemorrhages in the skin	No. of pigs with symptoms/No. of pigs observed	No. of pigs with symptoms/No. of pigs observed	No. of pigs with symptoms/No. of pigs observed
8	Neurological signs	No. of pigs with symptoms/No. of pigs observed	No. of pigs with symptoms/No. of pigs observed	No. of pigs with symptoms/No. of pigs observed
9	Coma	No. of pigs with symptoms/No. of pigs observed	No. of pigs with symptoms/No. of pigs observed	No. of pigs with symptoms/No. of pigs observed

### Statistical analysis

Statistical analyses were performed using the statistical software GraphPad Prism 5.01 (GraphPad Software, USA). Data were compared using the one-way repeated measure analysis of variance test. Normal distribution was checked visually from distributions and with Shapiro–Wilk’s W test. p≤0.05 was considered as significant. Results expressed as the mean and standard error of the mean.

## Results and Discussion

### Swine screening for known pathogens

Before starting the experiment, piglets were screened for pathogens, such as ASFV, FMDV, PRRSV, CSFV, and ASFV IgG. The experimental pigs were free of known pathogens to avoid any bias in the study.

### Appearance of clinical signs in the experimental pig groups

**Figure-1:**
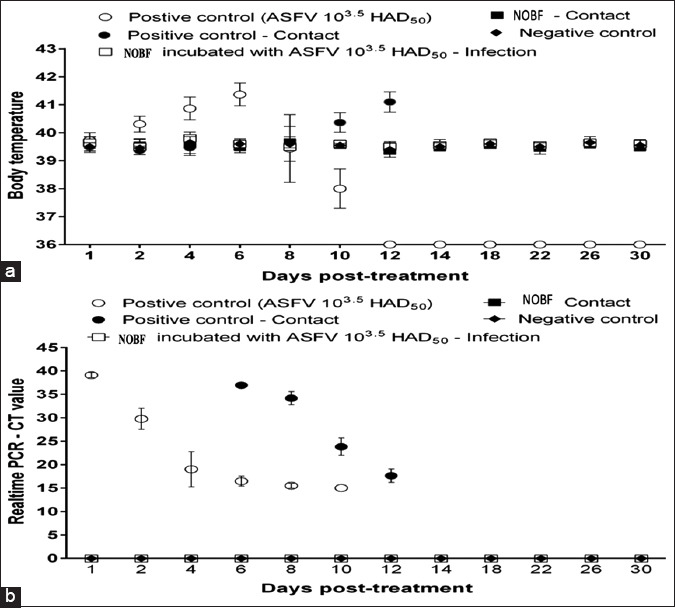
Body temperature and real-time polymerase chain reaction analysis of African swine fever virus replication in blood samples of experimental pigs in the natural oil blend formulation treatment 1 and treatment 2, and control groups in the in vivo trial.

All pigs from the incubated virus-challenged treatment, the co-housed pigs in the direct challenged treatment group, and the negative control group did not show any clinical symptoms or abnormalities. The clinical signs started 4-6 days post-infection in challenged pigs and at 10-12 days in co-housed pigs of the positive control. The clinical symptoms started at 14-16 days in co-housed pigs in the direct-challenge treatment group. The symptoms included high fever (≥41°C), diarrhea, vomiting, loss of appetite and reflexes, depression, respiratory distress, cyanosis on the edges of the ears, tail, and legs, hemorrhages in the skin, bleeding from the nose and rectum (1/5), neurological signs, coma, and death (Table-4 and Figure-1). Table-4 shows the typical symptoms of ASFV infection and these are in alignment with those of our previous studies [6-8], which appeared in the positive control pigs and the treatment groups’ challenged pigs. Remarkably, all contact pigs from the treatment group remained healthy.

**Table-4:** Clinical signs of VNUA-ASFV-L01/HN/04/19-inoculated pigs and transmission in experimental pigs.

No.	Clinical signs	No. of pigs in positive control	No. of pigs in treatment 1	No. of pigs in treatment 2	No. of pigs in negative control
1	High fever	6/6	3/6	0/6	0/3
2	Depression	6/6	3/6	0/6	0/3
3	Loss of appetite and reflexes	6/6	3/6	0/6	0/3
4	Vomiting	4/6	0/6	0/6	0/3
5	Respiratory distress	6/6	0/6	0/6	0/3
6	Cyanosis on the edges of the ears, tail and legs	5/6	2/6	0/6	0/3
7	Hemorrhages in the skin	5/6	2/6	0/6	0/3
8	Neurological signs	5/6	2/6	0/6	0/3
9	Coma	5/6	3/6	0/6	0/3

Lesions of the heart, lungs, gallbladder, spleen, mesenteric lymph nodes, and gastrohepatic lymph nodes of the pigs inoculated with 10^3^ HAD50 of ASFV were identified. Hemorrhagic lesions were typically observed in multiple tissues and organs, which is in accordance with the results of the previous studies [6-8].

The OIA guidelines [6-8] were followed to measure and report the temperature. A pig with a body temperature >40.50°C was considered to have a high fever [6-8]. In domestic swine, the incubation period ranged from 5-15 days. The clinical symptoms of ASF, previously reported by Galindo [1], include fever (41-42°C for approximately 4 days), diarrhea, inappetence, incoordination, prostration, coma, and death. Vomiting, nasal and conjunctival discharge, dyspnea, and anal and nasal hemorrhages can also be observed in some animals. The body temperature of infected pigs increased gradually up to a maximum (42°C) and then suddenly decreased to 37°C or below, followed by mortality. All inoculated pigs in the positive control and challenged pigs in the direct challenge treatment group exhibited high fever post-challenge. The body temperatures of all pigs of the incubated challenge group, cohoused pigs of the direct challenge treatment group, and pigs of the negative control group were average (Figure-1). The most interesting observation was that the body temperature of the highly infected pigs fell to 37°C before the individual collapsed and died.

### RT-PCR monitoring results

**Figure-2:**
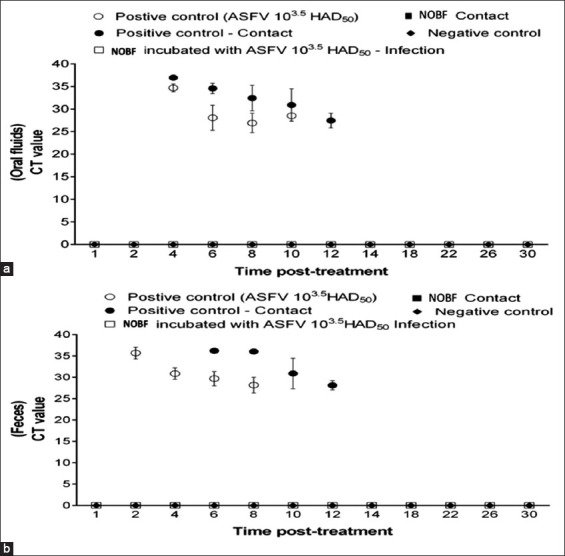
African swine fever virus (ASFV) shedding in oral fluid in experimental pigs post inoculation. (a) ASFV shedding in the feces of infected pigs post-treatment. (b) ASFV shedding in the feces of infected pig’s post-treatment.

VNUA-ASFV-L01/HN/04/19 replication was analyzed in the blood samples of experimental pigs at different time points post-treatment. The RT-PCR results showed that the blood samples of contact pigs from the treatment group were negative for ASFV (Table-5), but were positive with a gradually increasing viral copy number within days in the positive control and challenged treatment groups. There was a positive correlation between body temperature and virus replication (i.e., the higher the viral load and the higher the body temperature) in the blood samples among the groups (Table-5 and Figure-2). Similar correlations and findings were reported by Beltrán-Alcrudo *et al*. [19].

**Table-5:** Replication of ASFV in blood samples post-treatment. Checks were performed up to 20 days of challenge. Individual pig samples were collected to screen for viral presence.

Day of challenge/Tag no	Negative Control			Treatment 1 NOBF (Direct challenge)						Treatment 1 NOBF (Incubated challenge)						Positive control					
			
	No challenge			Challenge			Cohoused			Challenge			Cohoused			Challenge			Cohoused		
						
	45	46	47	100	73	81	76	77	79	50	51	52	53	54	55	67	89	96	72	75	97
D-0	ND	ND	ND	ND	ND	ND	ND	ND	ND	ND	ND	ND	ND	ND	ND	ND	ND	ND	ND	ND	ND
D-10	ND	ND	ND	ND	ND	ND	ND	ND	ND	ND	ND	ND	ND	ND	ND	ND	ND	ND	ND	ND	ND
D-12	ND	ND	ND	34.62	32.15	34.76	ND	ND	ND	ND	ND	ND	ND	ND	ND	27.56	29.67	32.06	ND	ND	ND
D-14	ND	ND	ND	30.91	20.85	21.66	ND	ND	ND	ND	ND	ND	ND	ND	ND	16.51	17.21	23.38	ND	ND	ND
D-16	ND	ND	ND	19.25	15.98	17.91	ND	ND	ND	ND	ND	ND	ND	ND	ND	15.41	16.23	17.52	ND	35.95	ND
D-18	ND	ND	ND	24.17	-	16.75	ND	ND	ND	ND	ND	ND	ND	ND	ND	15.03	15.19	16.05	35.22	33.56	34,83
D-20	ND	ND	ND	22.44	-	-	ND	ND	ND	ND	ND	ND	ND	ND	ND	-	-	15.03	24.52	23.19	27,83

Ct value≥37 negative real-time PCR; ≤36=Positive real-time PCR; ND (not detected)=Negative real-time PCR. ASFV=African swine fever virus, NOBF=Natural oil blend formulation

RT-PCR is the most advanced technique available for pathogen detection in the pig industry. It is highly sensitive and time-consuming. The OIE recommends quantitative PCR using RT-PCR primers and probes [19].

**Figure-3:**
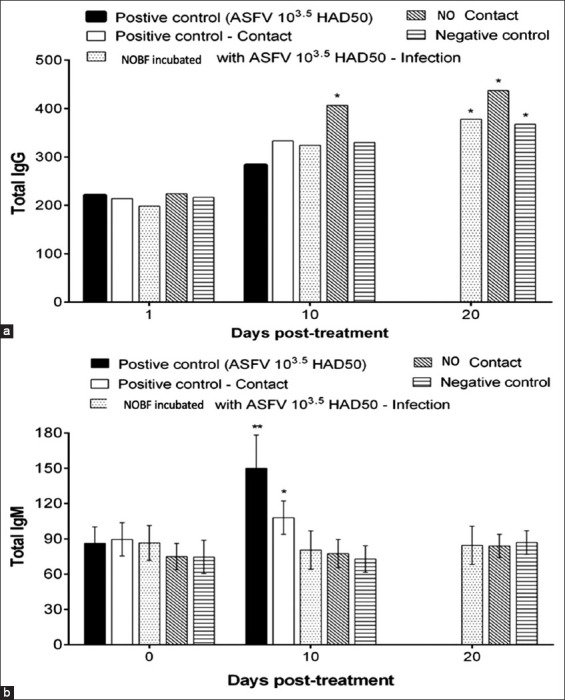
Analysis of total immunoglobulin G (IgG) and immunoglobulin M (IgM) levels in the serum samples of experimental pigs in the treatment groups. (a) Analysis of total IgG in the experimental pigs of the natural oil blend formulation (NOBF) and control groups; (b) Analysis of total IgM in the experimental pigs of the NOBF and control groups.

Samples of oral fluid and feces of pigs were tested by RT-PCR to confirm the viral shedding phenomenon responsible for horizontal transmission. The ASFV was detected in the oral fluid (day 14) and fecal samples (day 12) of the positive control group and challenged treatment group (Tables-6 and 7 and Figures-2a and b). In contrast, all the pigs in the incubated challenged treatment and the direct challenge treatment group cohoused pigs were negative (Figure-3a and b). The challenged pigs in the direct challenge treatment group showed positive results. The positive control and IM-challenged pigs shed the virus and infected the contact pigs horizontally. The results of the incubated challenge treatment showed that NOBF had strong antiviral properties. NOBF can inhibit the horizontal transmission of ASFV, which was observed in the cohoused pigs of the direct challenge treatment group. The challenged pigs in the direct challenge treatment group showed delayed infection, which showed that the given dose of NOBF was not strong enough to inhibit the multiplication of the ASFV. This suggests that NOBF is an efficient supplement to prevent pigs from contracting ASFV infections.

**Table-6:** ASFV shedding in oral fluid post-treatment.

Day of challenge/Tag no	Negative Control			Treatment 1 NOBF (Direct challenge)						Treatment 1 NOBF (Incubated challenge)						Positive control					
			
	No challenge			Challenge			Cohoused			Challenge			Cohoused			Challenge			Cohoused		
						
	45	46	47	100	73	81	76	77	79	50	51	52	53	54	55	67	89	96	72	75	97
D-0	ND	ND	ND	ND	ND	ND	ND	ND	ND	ND	ND	ND	ND	ND	ND	ND	ND	ND	ND	ND	ND
D-10	ND	ND	ND	ND	ND	ND	ND	ND	ND	ND	ND	ND	ND	ND	ND	ND	ND	ND	ND	ND	ND
D-12	ND	ND	ND	ND	ND	ND	ND	ND	ND	ND	ND	ND	ND	ND	ND	ND	ND	ND	ND	ND	ND
D-14	ND	ND	ND	ND	35.82	ND	ND	ND	ND	ND	ND	ND	ND	ND	ND	34.15	35.23	ND	ND	35.95	ND
D-16	ND	ND	ND	ND	29.53	31.41	ND	ND	ND	ND	ND	ND	ND	ND	ND	25.14	28.42	30.63	33.61	34.26	33.82
D-18	ND	ND	ND	ND	-	29.33	ND	ND	ND	ND	ND	ND	ND	ND	ND	25.03	25,55	26,39	29.27	34.76	33.25
D-20	ND	ND	ND	36.53	-	33.25	ND	ND	ND	ND	ND	ND	ND	ND	ND	-	-	-	27.07	33.26	32.64

Ct value≥37 negative real-time PCR; ≤36=Positive real-time PCR; ND (not detected)=Negative real-time PCR. ND=Not detected. ASFV=African swine fever virus, NOBF=Natural oil blend formulation

**Table-7:** ASFV shedding in fecal samples posttreatment. Checking was performed up to 20 days of challenge. Individual pig samples were collected to screen for viral presence.

Day of challenge/Tag no	Negative Control			Treatment 1 NOBF (Direct challenge)						Treatment 1 NOBF (Incubated challenge)					Positive control					
			
	No challenge			Challenge			Cohoused			Challenge			Cohoused		Challenge		Cohoused			
						
	45	46	47	100	73	81	76	77	79	50	51	52	53	54	55	89	96	72	75	97
D-0	ND	ND	ND				ND	ND	ND	ND	ND	ND	ND	ND	ND	ND	ND	ND	ND	ND
D-10	ND	ND	ND				ND	ND	ND	ND	ND	ND	ND	ND	ND	ND	ND	ND	ND	ND
D-12	ND	ND	ND				ND	ND	ND	ND	ND	ND	ND	ND	ND	35.89	36.92	ND	ND	ND
D-14	ND	ND	ND				ND	ND	ND	ND	ND	ND	ND	ND	ND	30.72	32,26	ND	ND	ND
D-16	ND	ND	ND				ND	ND	ND	ND	ND	ND	ND	ND	ND	29.29	31.52	36.21	ND	ND
D-18	ND	ND	ND				ND	ND	ND	ND	ND	ND	ND	ND	ND	27.31	30.26	35.93	36.1	ND
D-20	ND	ND	ND				ND	ND	ND	ND	ND	ND	ND	ND	ND	-	29.23	35.17	35.26	36.03

Ct value≥37 negative real-time PCR; ≤36=Positive real-time PCR; ND (not detected)=Negative real-time PCR. ASFV=African swine fever virus, NOBF=Natural oil blend formulation

### Protection level

The VNUA-ASFV-L01/HN/04/19 virus strain was isolated from pigs with apparent symptoms in the Thai Binh Province, Vietnam. The virus strain was purified and quantified at the Molecular Biology Laboratory of VNUA, Vietnam. The lethal and sublethal doses were optimized under controlled conditions. The log3 quantity of ASFV was considered a sublethal dose that could kill pigs within 7-10 days after the challenge.

The ASFV-challenged pigs from the positive control group died at 8-10 days post-challenge. The cohoused pigs from the positive control group died at 14-16 days post-challenge. The incubation period of the disease varied at 4-19 days, depending on the number of viral copies present in the body and virulence [19]. The acute stage of ASF was characterized by a high fever, with a mortality rate of up to 100% within 9 days of infection with typical symptoms, such as anorexia, lethargy, inactivity, bunching up together [19], and hemorrhagic spots on the ears, abdomen, and hind legs, generalized reddening of the skin, bleeding from the nose/mouth, and bloody feces at a later stage [16].

The essential oils present in NOBF have antiviral properties that can deactivate the ASFV [2,20]. The antiviral activity of all the essential oils tested was demonstrated for enveloped viruses. Essential oils affect the viral envelope, which is necessary for adsorption or entry into the host cells. In particular, monoterpenes showed increased cell membrane fluidity and permeability, altering the membrane protein order [20]. Lavender essential oil consists primarily of monoterpenoids and sesquiterpenoids. Among these, linalool dominates, with moderate levels of lavandula acetate, terpinene-4-ol, and lavandulol. 1,8-Cineole and camphor are also present at low-to-moderate quantities. Lavender oil may be useful for alleviating anxiety and sleep disorders in infected pigs. It also has antimicrobial, anti-inflammatory, and mood-alleviating effects [20,21]. Pine oil mainly consists of alpha-terpineol or cyclic terpene alcohols. It may also contain terpene hydrocarbons, ethers, and esters. Pine oil is a phenolic disinfectant that is mildly antiseptic and has antifungal, antibacterial, and antiviral properties [19,21]. Eucalyptus oil has wide applications as a pharmaceutical, antiseptic, repellent, flavoring, and fragrance as well as for industrial use; in the British Pharmacopeia, it must contain a cineole minimum of 70%. Eucalyptus oil has antibacterial, antiviral, and anti-inflammatory effects. Preclinical results also show that eucalyptus oil stimulates the innate cell-mediated immune response by affecting human monocyte-derived macrophages [20-22]. Eucalyptus oil shows potential antiviral activity against herpes and yellow fever viruses. Its activity has also been established against viral envelope structures [23].

### Immune response induced by NOBF in experimental pigs during treatment

Essential oils possess immunomodulatory properties that affect both the cellular and molecular levels of the immune system of animals [23]. ASF causes immunodeficiency in the affected pigs [12]. The virus initially replicates in the tonsils or regional lymph nodes [24]. Blood and lymph play roles as secondary locations of replication in viral spread [25]. The ASFV mainly targets monocytes and macrophages, which are responsible for the HAD property of HAD isolates that cause acute disease compared to non-HAD isolates [26].

**Figure-4:**
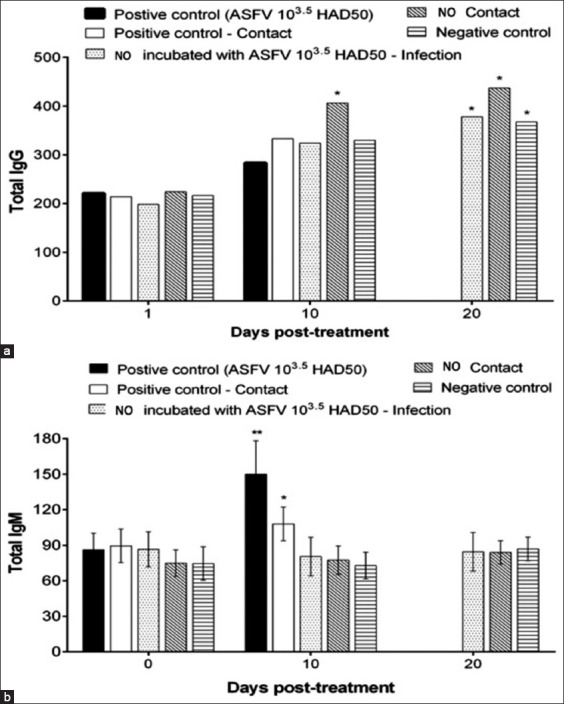
Analysis of total immunoglobulin G (IgG) and immunoglobulin M (IgM) levels in the serum samples of experimental pigs in the treatment groups. (a) Analysis of total IgG in the experimental pigs of the natural oil blend formulation (NOBF) and control groups; (b) analysis of total IgM in the experimental pigs of the NOBF and control groups.

Analysis of serum samples from experimental pigs from all groups showed that the use of NOBF could reduce the mucosal immune response characterized by slightly higher IgG levels and lower IgM levels in the NOBF groups than in the positive control group (Figures-4a and b) The levels of IgA and interferon-gamma (IFN-g) were also tested for all treated groups; however, no significant differences in IgA and IFN-g levels were observed (data not shown). The ASFV replicates in macrophages and monocytes [26,27]. Lymphopenia and neutrophilia are commonly observed during ASFV infection. The viral pathogenesis may be due to cytokines produced by infected monocytes and macrophages [28,29]. Higher levels of IgG and lower levels of IgM [30] in the NOBF-treated groups resulted in a higher level of protection than that in the positive control, which explains the immunomodulatory effect of NOBF. The mechanism of immune enhancement can work in several ways by promoting the activity of lymphocytes, increasing phagocytosis by macrophages, inducing IFN production, or by stimulating NK cell activity. Eucalyptus oil, with its major component of 1,8-cineole, stimulates phagocytic activity [23]. Lavender oil reportedly increases the phagocytic rate and represses major pro-inflammatory cytokines, exerting an anti-inflammatory effect [31]. Pine bark extract can reduce the number of pathogens in raw beef as a potent antibacterial agent, free radical scavenger, and effective enzyme inhibitor [32]. The positive effects on cellular and humoral immune responses in animals have been reported due to the presence of proanthocyanidins in pine extract [33].

The blend of these oils enhances the innate immune response by stimulating phagocytosis [31]. The essential oil blend supplementation improved the immune status (IgA, IgM, and IgG) in weaned pigs [34]. The oil blend supplement mainly contained linalool, thymol, and cinnamaldehyde [24,25,35-38], which is similar to the composition of NOBF.

### Blood physical parameters of experimental pigs

Analysis of the physical blood parameters (22 parameters) of the ASFV-NOBF incubation group in either inoculated or contact pigs revealed that the use of NOBF did not affect physical blood parameters. The values of physical blood parameters were similar to those of the negative and positive control groups (Tables-8 and 9). Significant differences in platelets (PLT) values, procalcitonin (PCT), granulocyte (GRAN) %, lymphocyte %, monocyte %, lymphocyte levels, and monocyte levels were found between the treatment and positive control groups. This demonstrated that pigs infected with virulent ASFV had reduced platelet counts due to hemorrhage in multiple organs. PLT help stop bleeding, particularly in endothelial blood vessels, and considerably decrease platelet levels, leading to internal bleeding in the brain and the quick death of pigs. In addition, the numbers of lymphocytes and monocytes in pig blood were significantly decreased due to ASFV infection. The damage and hemorrhage in multiple organs caused by the ASFV led to immune system effects, especially the phagocytosis of pathogens, leading to a decrease in the protective ability against pathogens. Similar results were observed when essential oil treatment inhibited 5-F-induced myelotoxicity and restored GRAN, monocyte, and monocyte-derived macrophase phagocytic ability [23,39].

**Table-8:** Blood physical parameters of pigs inoculated with ASFV treated with NOBF.

Blood physical parameters	Parameter	ASFV incubated with NOBF- Infected pigs (mean)						NOBF – Cohoused pigs (mean)					
	
		D0	D2	D4	D6	D8	D10	D0	D2	D4	D6	D8	D10
Red Blood Count (RBC)	T/L	5.74	5.50	5.83	6.88	5.94	6.25	5.45	5.42	5.36	5.64	5.19	5.24
Hemoglobin (Hb)	g/dL	10.37	9.63	9.79	11.90	10.23	10.80	9.87	9.55	9.01	9.58	9.30	8.90
Hematocrit (HCT)	%	34.43	31.57	31.75	38.30	35.17	37.23	32.13	31.05	29.15	31.40	31.60	30.55
Mean Corpuscular Volume (MCV)	fL	60.00	57.33	54.45	55.75	59.00	59.67	60.00	57.00	54.50	55.70	57.00	58.00
Mean Corpuscular hemoglobin (MCH)	pg	18.03	17.53	16.75	17.35	17.23	17.30	18.35	17.60	16.85	17.00	16.35	17.00
Mean Corpuscular hemoglobin concentration(MCHC)	g/dL	30.07	30.57	30.80	31.15	29.10	29.03	30.55	30.70	30.95	30.50	28.65	29.10
Red blood cell distribution width (RDW-CV)	%	19.70	18.83	22.80	22.95	18.33	17.90	19.00	17.45	20.25	20.70	17.20	17.10
Platelets (PLT)	G/L	378	422	396	474.5	379	382.67	416	474	501.5	467.5	323.5	425.5
Mean platelet volume (MPV)	fL	9.27	9.17	10.85	9.52	8.43	9.73	8.87	9.70	10.11	9.70	10.75	10.60
Procalcitonin (PCT)	%	0.36	0.38	0.42	0.39	0.35	0.33	0.37	0.46	0.41	0.39	0.35	0.45
Platelet distribution width (PDW)	%	13.47	12.87	13.21	11.68	10.93	10.97	11.93	11.70	12.04	10.32	9.40	12.26
White Blood count (WBC)	G/L	16.90	23.43	17.25	5.08	20.73	21.50	17.60	34.70	21.55	4.34	21.25	12.65
Granulocyte (GRAN) %	%	29.97	32.93	37.00	95.20	31.53	34.00	29.47	34.95	29.50	90.50	22.50	29.15
Lymphocyte %	%	53.23	56.27	56.45	0.00	59.50	53.87	56.30	52.85	66.65	0.00	67.75	28.30
Monocyte %	%	16.80	10.80	3.29	0.00	8.97	12.13	14.23	12.20	0.75	0.00	9.75	58.85
Absolute Neutrophil count (ANC)	G/L	5.00	7.83	6.42	4.88	6.70	6.25	5.00	12.10	6.85	3.93	4.65	12.85
Lymphocytes	G/L	9.07	13.13	9.75	0.00	12.20	11.37	10.17	18.40	14.03	0.00	14.55	8.20
Monocytes	G/L	2.83	2.47	0.55	0.00	1.83	2.57	2.43	4.20	0.19	0.00	2.05	17.20
Eosinophil count test (EOS) %	%	0.57	0.57	0.57	0.30	0.57	0.57	0.44	0.41	0.46	0.41	0.39	0.45
BASO %	%	0.00	0.00	0.01	0.00	0.01	0.00	0.00	0.01	0.00	0.00	0.00	0.01
Eosinophil %	%	4.23	4.36	5.23	4.81	3.23	4.91	4.70	3.83	6.73	5.51	4.97	5.86
Basophil %	%	0.00	0.02	0.03	0.00	0.00	0.01	0.00	0.03	0.00	0.00	0.02	0.00

ASFV=African swine fever virus, NOBF=Natural oil blend formulation

**Table-9:** Blood physical parameters of negative and positive control pigs.

Blood physical parameters	Parameter	Negative control pigs (mean)						Positive control - challenged pigs (mean)						Positive control -Cohoused pigs (mean)					
		
		D0	D2	D4	D6	D8	D10	D0	D2	D4	D6	D8	D10	D0	D2	D4	D6	D8	D10
Red Blood Count (RBC)	T/L	5.785	6.315	6.13	6.005	5.77	5.765	6.16	5.71	6.31	5.59	5.09	4.81	5.39	4.55	5.00	4.47	4.60	5.54
Hemoglobin (Hb)	g/dL	10.25	10.7	10.7	10.4	9.6	9.6	10.27	8.93	9.70	8.89	7.75	7.80	8.80	7.27	7.88	6.87	6.90	8.45
Hematocrit (HCT)	%	33.2	36.4	34.4	35.55	32.8	32.75	34.23	30.10	30.87	27.37	25.70	25.70	30.00	23.90	24.27	21.93	24.00	28.95
Mean Corpuscular Volume (MCV)	fL	57.5	57.85	56.25	59	57	57	55.33	52.67	48.83	49.10	50.50	54.00	56.00	52.67	48.90	49.73	53.00	53.00
Mean Corpuscular hemoglobin (MCH)	pg	17.7	17	17.45	17.45	16.7	16.65	16.67	15.70	15.37	15.97	15.20	16.20	16.53	16.10	15.87	15.57	15.30	15.40
Mean Corpuscular hemoglobin concentration(MCHC)	g/dL	30.8	29.4	31	29.35	29.3	29.25	30.10	29.77	31.47	32.47	30.20	30.20	29.43	30.57	32.50	31.33	28.95	29.15
Red blood cell distribution width (RDW-CV)	%	18	19.45	19.6	16.7	17.2	17.9	19.33	18.73	22.17	23.73	19.15	18.80	21.07	20.37	23.40	23.97	22.30	23.00
Platelets (PLT)	G/L	541	534.5	517.5	430.5	411	410.5	377	312	216.67	190.73	132.5	89.00	485.0	443.67	418	428.1	452	308
Mean platelet volume (MPV)	fL	8.25	11.51	8.76	9.55	10.1	9.20	9.00	9.47	10.30	11.60	9.65	9.60	9.03	11.53	10.50	14.10	9.70	10.00
Procalcitonin (PCT)	%	0.42	0.36	0.31	0.29	0.34	0.335	0.34	0.29	0.21	0.18	0.13	0.10	0.44	0.42	0.53	0.51	0.46	0.41
Platelet distribution width (PDW)	%	11.5	11.62	10.19	9.62	9.25	9.25	10.97	9.63	10.60	11.20	12.95	11.00	12.30	9.63	10.90	11.15	10.20	12.85
White Blood count (WBC)	G/L	24.75	20.3	19.1	19.4	19.8	19.75	17.50	34.47	23.20	8.93	19.90	13.80	19.13	21.33	18.59	26.03	27.85	26.70
Granulocyte (GRAN) %	%	36.5	38.25	30.40	38.9	32.9	32.9	31.40	47.97	61.37	88.25	57.00	64.10	34.03	38.13	33.00	66.80	45.35	49.25
Lymphocyte %	%	49.52	56.55	50.29	51.75	55.2	55.15	67.43	49.40	52.77	50,21	46.35	33.70	50.37	52.90	48.73	0.00	45.10	45.15
Monocyte %	%	13.5	12.87	11.64	10.35	12	11.95	11.17	12.63	8.60	7.06	6.65	2.20	12.60	8.97	9.28	8.16	9.55	10.60
Absolute Neutrophil count (ANC)	G/L	9.35	7.975	7.715	7.65	7.82	7.82	6.73	16.60	13.33	8.60	7.29	8.85	5.90	8.18	7.41	5.77	12.30	12.10
Lymphocytes	G/L	11.75	11.35	11.66	10.93	10.9	10.85	11.90	12.53	7.11	6.58	8.25	4.65	9.60	11.23	8.86	10.04	12.10	12.60
Monocytes	G/L	3.62	2.91	2.86	2.75	2.65	2.65	2.87	4.03	2.37	2.01	1.36	0.40	2.63	2.52	1.95	2.18	2.45	2.95
Eosinophil count test (EOS) %	%	0.40	0.44	0.374	0.42	0.39	0.39	0.382	0.41	0.20	0.31	0.38	0.36	0.31	0.29	0.23	0.35	0.34	0.27
BASO %	%	0.01	0.00	0.01	0.00	0.00	0.00	0.02	0.00	0.03	0.00	0.02	0.01	0.00	0.01	0.02	0.00	0.01	0.01
Eosinophil %	%	3.28	2.25	5.23	5.18	5.29	4.83	2.72	3.16	4.11	3.72	3.36	3.58	2.93	5.16	3.91	3.20	8.83	5.62
Basophil %	%	0.04	0.034	0.03	0.00	0.00	0.00	0.00	0.02	0.07	0.10	0.06	0.05	0.04	0.00	0.03	0.00	0.02	0.00

Blood cell counts showed lymphopenia, monocytopenia, and neutrophilia during acute and subacute infections [26]. Although the molecular mechanisms of viral hemorrhagic fevers are not well known, it is believed that a “cytokine storm” due to excessive pro-inflammatory cytokine responses plays an essential role in pathogenesis [28,29].

ASF pathogenesis may mainly be due to cytokines produced by infected monocytes and macrophages, but the molecular basis of ASF pathogenesis is not currently well-understood [28,29]. The lymphoid organs, including the spleen, lymph nodes, thymus, and tonsils, are destroyed in the acute form [30], and significant populations of B and T lymphocytes and macrophages undergo cell death [21], which correlates with the results of the present study (Tables-8 and 9).

The antimicrobial effects of essential oils are linked to their composition and cytotoxic effects, which cause cell membrane damage. These compounds are lipophilic and pass through the cell wall and the cytoplasmic membrane. Farm biosafety measures are critical. Farm laborers handling infected pigs should take all precautions to avoid the chances of horizontal transmission of the ASFV [19,40]. Practices to prevent ASFV infection through natural methods and by strengthening biosecurity measures will help control disease spread and outbreaks, especially as no successful vaccine agents are currently available.

## Conclusion

In this study, we attempted to demonstrate that NOBF can deactivate the ASFV during direct contact and incubation, prevent horizontal transmission, and minimize the risk of ASFV contamination in a population.

The NOBF incubation group showed that the ASFV was deactivated by incubation in NOBF solution, as proven by RT-PCR, mortality rates, and blood analysis. We also proved the concept of NOBF as an anti-ASFV agent in an *in vitro* trial reported previously. The second group showed that NOBF prevented horizontal transmission from injected or challenged pigs to contact pigs. The contact pigs of the treatment group who shared a water tap, living space, and feed tray, among other resources, with the challenged pigs did not become infected with the ASFV. The pigs were negative by RT-PCR, and no clinical signs of the ASFV were observed.

The clinical signs of the ASFV and mortality occurred in IM-challenged pigs in the treatment group but were slower than in the positive control group. NOBF showed a level of protection, but it was not sufficient to degenerate the virus in the pig body. Future studies should aim to determine and optimize the dose to treat ASFV-infected pigs. NOBF has an immune function along with the direct antiviral properties that balance the immune properties.

The trial outcomes can be summarized as follows: (1) NOBF can deactivate the ASFV by co-incubation and (2) prevent its horizontal transmission. (3) NOBF is a suitable water supplement candidate for ASFV infection prevention. (4) NOBF can deactivate the ASFV in the environment, especially in the saliva and feces of infected pigs, thereby preventing horizontal transmission in a population. (4) NOBF is well-tolerated and safe for use in animals.

## Authors’ Contributions

YB, HYB, QLT, and RKJ: Contributed to the study conception. HTN: Contributed to sample analysis. HYB, QLT, and RKJ: Designed and conducted the experiments. HYB, QLT, and RKJ: Analyzed the data. HYB and RKJ: Drafted the manuscript. YB, AA, LTN, and TLT: Edited the manuscript. All authors read and approved the final manuscript.
